# Factors associated with cutaneous ulcers among children in two yaws-endemic districts in Ghana

**DOI:** 10.1186/s40249-020-00641-2

**Published:** 2020-03-12

**Authors:** Rafiq Nii Attoh Okine, Bismark Sarfo, Richard M. Adanu, Cynthia Kwakye-Maclean, Francis Adjei Osei

**Affiliations:** 1 World Health Organization (WHO), Country Office for Ghana, Accra, Ghana; 2 Department of Epidemiology and Disease Control, School of Public Health, College of Health, Sciences, University of Ghana, Accra, Ghana; 3 Department of Population, Family and Reproductive Health, School of Public Health, College of Health, Sciences, University of Ghana, Accra, Ghana; 4 National Program Office, National Yaws Eradication Program (NYEP), Accra, Ghana; 5 Public Health Unit, Komfo-Anokye Teaching Hospital, Kumasi, Ghana; 6 KNUST School of Public Health, Kumasi, Ghana

**Keywords:** Yaws, Dual path platform, Upper west Akyem, Awutu Senya west, Children

## Abstract

**Background:**

Yaws is a chronic relapsing disease caused by *Treponema pallidum* subspecies *pertunue*, which can result in severe disability and deformities. Children below the age of 15 years in resource-poor communities are the most affected. Several non-specific factors facilitate the continuous transmission and resurgence of the disease. Endemic communities in rural Ghana continue to report cases despite the roll out of several intervention strategies in the past years. The objective of this study was to determine the factors associated with cutaneous ulcers among children in two yaws-endemic districts in Ghana.

**Methods:**

A community-based unmatched 1:2 case-control study was conducted among children between 1 and 15 years. Data on socio-demographic, environmental and behavioral factors were collected using a structured questionnaire. Active case search and confirmation was done using the Dual Path Platform (DPP) Syphilis Screen and Confirm test kit. Data were analyzed using STATA 15. Logistic regression was done to determine the exposures that were associated with yaws infection at 0.05 significant level.

**Results:**

Sixty-two cases and 124 controls were recruited for the study. The adjusted multivariable logistic regression model showed that yaws infection was more likely among individuals who reside in overcrowded compound houses (a*OR* = 25.42, 95% *CI:* 6.15–105.09) and with poor handwashing habits (a*OR* = 6.46, 95% *CI:* 1.89–22.04). Male (a*OR* = 4.15, 95% *CI:* 1.29–13.36) and increasing age (a*OR* = 5.90, 95% *CI:* 1.97–17.67) were also associated with yaws infection.

**Conclusions:**

Poor personal hygiene, overcrowding and lack of access to improved sanitary facilities are the factors that facilitate the transmission of yaws in the Awutu Senya West and Upper West Akyem districts. Yaws was also more common among males and school-aged children. Improving living conditions, access to good sanitary facilities and encouraging good personal hygiene practices should be core features of eradication programs in endemic communities.

## Background

Yaws is one of the three non-venereal treponemal diseases that is considered as a neglected tropical disease (NTD) [1]. The disease is caused by *Treponema pallidum* subspecies *pertenue.* It is a highly contagious disease which has a chronic relapsing pattern [2]. Yaws is transmitted through skin-to-skin contact and primarily affects children under the age of 15 years typically in areas of low socio-economic status and sanitation challenges. The disease is endemic in low-to-middle income countries and resurgence has been reported in the Pacific region, West and Central Africa and Southeast Asia [3]. The disease is usually self-limiting but can lead to severe complications involving the skin and bones. Typically, this results in destructive lesions of bones and cartilages and results in severe deformities and disability [4].

Despite concerted efforts and strategies to eradicate yaws, the disease is still endemic in at least fourteen countries [1]. A systematic review published in 2015, estimated that over 80 million people were living in endemic districts [2]. Over 250 000 cases of yaws were reported between 2010 and 2013 to the World Health Organization (WHO) from endemic countries [2]. Eighty-four (84) percent of the cases were from Ghana, Papua New Guinea and Solomon Island [2].

Ghana is among the countries in sub-Saharan Africa with increased prevalence of yaws. There are case reports of yaws in all the 16 regions with 160/170 health districts reporting cases [2]. In the last 8 years, averagely, 111 cases per 100 000 population/year were reported in the country [5]. Rural communities in Ghana continue to report new cases despite variety of strategies that have been implemented over the past years. A national school-based survey reported a prevalence of 20% amongst school children [6]. The Central, Eastern and Volta Regions remain endemic, reporting between 5.0–338 cases/100 000 population over a 4-year period [5].

WHO launched the Morges Yaws Eradication Strategy in 2012, with a target of eradication by 2020. A major component of the new strategy was the change from the injectable benzathine penicillin to oral azithromycin [7]. The new treatment strategy was piloted in three countries including Ghana [8–10] and though the strategy had significant impact on disease burden, it did not achieve elimination targets in high endemic communities [11]. This is a major pointer to further explore the environmental drivers of yaws transmission in highly endemic communities.

The National Yaws Eradication Program (NYEP) in Ghana, which was set up in 2008 has the singular task of co-coordinating all yaws activities in the country through collaboration with international agencies. The program had set elimination targets by the end of 2017 (zero indigenous cases from a baseline of 29 cases/100 000 population/year over the last 5 years) and total eradication by 2020 [12]. Ghana is likely to miss these targets due to multiplicity of factors including resource limitation to scale up integrated interventions and gaps in the understanding of the disease transmission process accounting for resurgence of the disease in some endemic districts.

Myriad of factors are known to be associated with yaws transmission. Personal factors (age, sex and cultural beliefs), behavioral factors (such as personal hygiene, sharing bathing items and contact with infected persons), environmental factors (unimproved toilet facilities, poor housing, and overcrowding) and lack of access to prompt diagnostic tools are among the factors that have been postulated to facilitate the disease transmission [13, 14].

Several studies have shown spatial heterogeneity and clustering of the disease in endemic communities [15]. There is also evidence that a good proportion of non-genital ulcerative skin lesions in yaws endemic communities have multiple causative agents including *Haemophilus ducreyi* [16, 17]. This calls for more research in understanding the epidemiology of the disease, particularly, the transmission process. There is currently no vaccine for yaws and the success of the eradication strategy is strongly linked to understanding the epidemiology of the disease, particularly the risk factors, since humans are the only known reservoirs. This study assessed the factors associated with cutaneous ulcers among children in two yaws-endemic districts in Ghana.

## Methods

### Study design

The study was a community-based unmatched 1:2 case-control study conducted in the Upper West Akyem and Awutu Senya West districts in the Eastern and Western Regions of Ghana respectively. The cases and controls were selected from households and schools within the same communities.

### Study areas

The two selected districts for the study continue to record high numbers of cases of yaws despite numerous interventions. The Awutu Senya West and Upper West Akyem districts are located in the Central and Eastern Regions respectively. The two districts share a common boundary, with the Upper West Akyem district located at the northeastern border of the Awutu Senya West district. The total household population of both districts is a little over 80 000 according to the 2010 population and housing census with children under the age of 15 years constituting more than 50% of the population.

High temperatures and humidity coupled with occasional heavy rainfall facilitate the transmission of yaws and other tropical diseases. The majority of the people live in rural areas, 52% in the Awutu Senya West district and 75% in the Upper West Akyem district [18].

Overcrowding is a major problem in both districts. Single room occupancy is as high as 91% in the Upper West Akyem district with more than 10% of such rooms accommodating more than 10 persons. The average number of persons per house in both districts is seven [18].

Public toilet facilities are commonly used in both districts and a significant number of the populace engage in open defecation. More than 40% of residents in the rural communities of both districts use open bathing cubicles.

A significant proportion of residents (about 20%) in both districts lack access to potable water and resort to rivers and streams as their main source of water for drinking and other household chores. Mud brick/earth (32.1%) is the second most important material used for outer walls of dwelling units in Awutu Senya West district. The proportion is slightly higher in the Upper West Akyem district (52.7%) [18].

### Study population

Children between ages 1–15 years who met the criteria for cases or control and resident in the Awutu Senya West and Upper West Akyem districts.

#### Case definition

The cases included in the study were cases with early or active yaws. Any child between the ages of 1–15 years who is resident in the Upper West Akyem or Awutu Senya West district who met the following criteria:

#### Suspected case

**Fig. 1:**
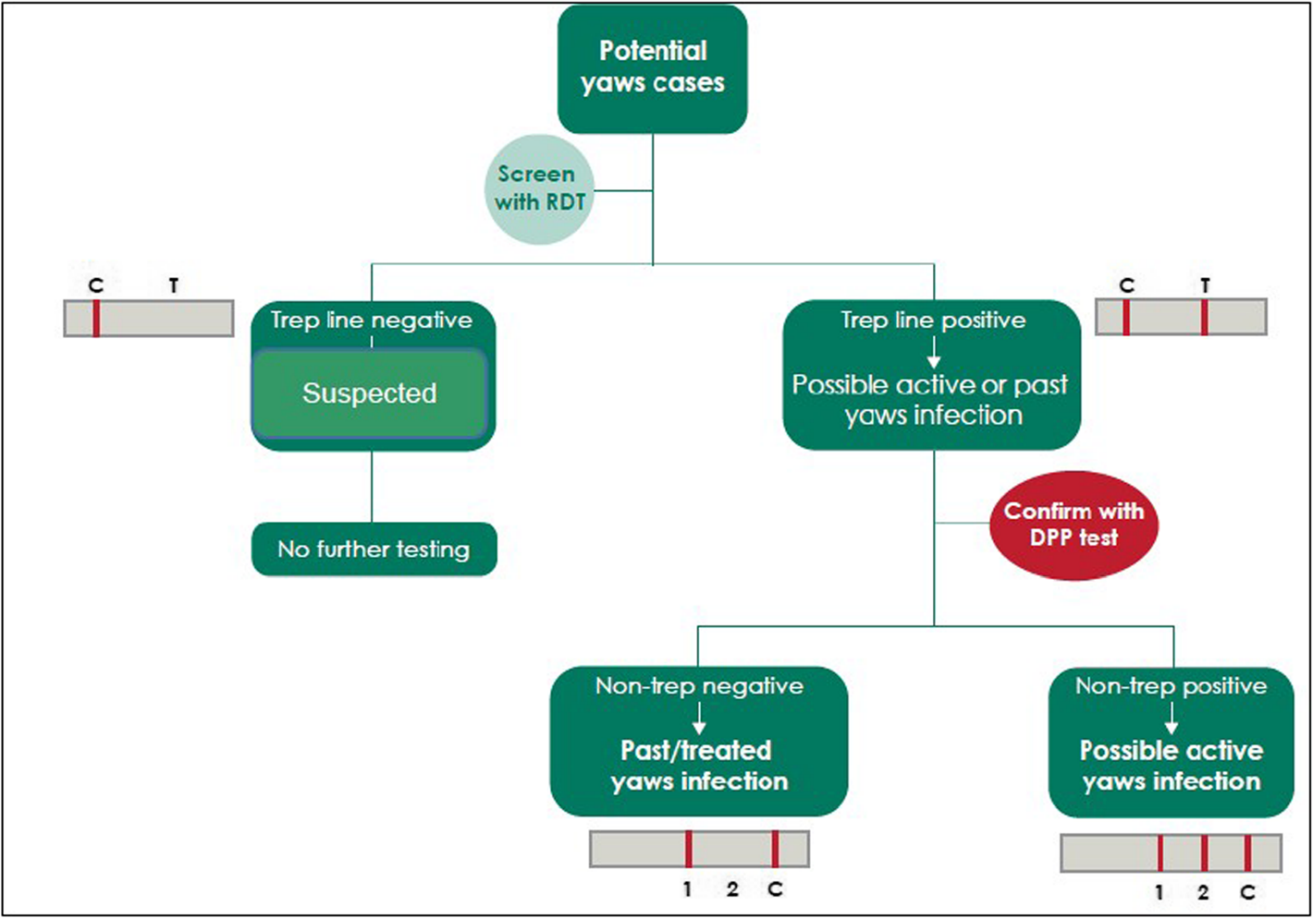
Algorithm for case selection (adapted from the World Health Organization guidelines)

Presence of primary and secondary lesions: papillomata, painless ulcer or hyperkeratosis. The cases were examined and selected using the WHO picture guide (Fig. 1).

Primary Yaws lesions: 1. Ulcers with raised edges and a crusty base or 2. Papilloma that appears as a firm yellowish skin lesion with a dark tip, on any part of the body.

Secondary Yaws lesions: Multiple ulcers or papillomata as previously defined, hyperkeratosis, macules, papules, nodules and maculopapular rash.

#### Probable case

A probable case was defined as any suspected case with only a positive rapid treponemal test. This test was done using the SD Bioline Syphilis 3.0® RDT.

#### Confirmed case

A probable case with a dually positive treponemal and non-treponemal test. Confirmation was done with rapid point of care (POC) test; The Dual Path Platform (DPP) Syphilis Screen and Confirm test kit (Chembio, Medford, NY, USA). The test kit has been shown to be highly specific and sensitive (95.2 and 92% respectively) compared to traditional *Treponemal* assays [19]. Finger prick blood samples were used for the test. The DPP tests results were read qualitatively and independently by two trained field officers. In instances where the two results were conflicting, a third independent reading by a senior field officer was taken as conclusive.

#### Controls

Children aged between 1 and 15 years in the Awutu Senya West and Upper West Akyem districts without a history of yaws or yaws-like skin lesions and living in the same neighborhood as the cases.

### Sample size and data collection

The total sample size for the study was 186 (62 cases and 124 controls) calculated assuming a power of 80% to detect an odds ratio of 3.0 with 18% exposure among controls [20] at an alpha level of 0.05 using OpenEpi Version 3 [21].

Data on exposures among cases and controls were collected using a pre-coded structured interviewer-administered questionnaire designed using the Research Electronic Data Capture (REDCap®) data capture tools hosted at the University of Ghana [22]. The questionnaire assessed the socio-demographic, environmental and behavioral factors associated with the transmission of yaws. Previous yaws infection was ascertained verbally from caregivers.

#### Case selection

Trained research assistants, field officers and the principal investigator identified cases from communities and schools in the district through active search using the criteria above.

#### Controls

Controls were selected from the same communities as cases. Two controls were selected for each case. A cardinal point, that is, east, west, north or south was selected through balloting in the house of an identified case. The first two houses in the balloted direction were then selected. A control was then selected in each house. Numbers were assigned to all children who met the criteria for controls in the house. A number was selected through balloting and the participant was included in the study. If no eligible controls were found in a selected house, the next house in the same direction was selected and the process repeated.

Controls were also selected in schools in instances where cases were identified in schools. The controls were selected from the same class where the cases were found. The selection was done through balloting, all eligible controls in the identified class were included in the balloting process. The number of controls subsequently selected was based on the number of cases identified in the class using a case to control the ratio of 1:2.

### Data processing and analysis

All data analysis was done with Stata 15 statistical software (StataCorp. 4905 Lakeway Drive Station, Texas 77 845, USA). Descriptive statistics were performed for all variables and expressed as means and standard deviation for continuous variables. Categorical variables were expressed as proportions and presented as graphs or charts where appropriate.

Univariable analysis (logistic regression) was done to test the association between socio-demographic factors, behavioral factors and yaws infection. This was presented as crude odds ratios with a 95% confidence interval. The final age and sex-adjusted multivariable logistic regression model fitted to determine the factors associated with yaws was done using backward stepwise approach (exposure variables with *P* values ≤ 0.1 were added and those with *P* values ≥ 0.2 were removed).

The analysis was done in two stages, initially with all cases (suspected, probable and confirmed) considered as a single group and then subsequently with only confirmed cases. The results description presented below represents all cases (suspected, probable and confirmed).

All statistical analysis was done at a 95% significance level with *P* values < 0.05 considered as statistically significant.

## Results

### Demographic characteristics of respondents

One hundred and eighty-six participants were recruited in the two districts, comprising of 62 cases and 124 controls.

The age range of the study participants was between 3 and 15 years. Cases were older than controls. The median age of cases was 11 years and the median age of controls was 10 years. There was however no statistically significant difference between the ages (*P* > 0.05). There was no significant statistical difference between the ages of males and females. Majority of the study participants in both districts were between the ages of 10–15 years, constituting more than half of participants in each district (Table 1).

**Table 1:** Age and sex distribution of study participants

	Controls (*n* = 124)	Cases (*n* = 62)	*P*-value	
Age (median, IQR)	10 (8, 13)	11 (9, 13)	0.076	
Sex (*n*, %)				
Male	64 (51.6)	46 (74.2)	0.003^ǂ^	
Female	60 (48.4)	16 (25.8)		
	Awutu Senya West	Upper West Akyem		
Age group	*n* (%)	*n* (%)	Total	*P*-value
< 5 years	3 (50.0)	3 (50.0)	6	0.646
5–9 years	29 (45.3)	35 (54.7)	64	
10–15 years	61 (52.6)	55 (47.4)	116	
	Males	Females		
Age group	N (%)	N (%)	Total	*P*-value
< 5 years	2 (33.3)	4 (66.7)	6	0.222
5–9 years	42 (65.6)	22 (34.4)	64	
10–15 years	66 (56.9)	50 (43.1)	116	

^ǂ^*P* <  0.05 means statistically significant

### Type and distribution of yaws lesions

Thirty-one cases were identified from each district. Half of the cases (50.0%, 31/62) were classified as suspected cases, 4 cases (6.5%) were classified as probable cases and 27 (43.6%) were confirmed using the DPP rapid test kit (Table 2).

**Table 2:** Yaws case classification and distribution in Upper West Akyem and Awutu Senya West districts

Variables	Case category, *n* (%)			
	Suspected	Probable	Confirmed	Total
Name of district				
Awutu Senya West	15 (48.4)	4 (100)	12 (44.4)	31
Upper West Akyem	16 (51.6)	0(0)	15 (55.6)	31
Type of lesion				
Ulcer	28 (90.3)	3 (75)	11 (40.7)	42 (67.7)
Papilloma	2 (6.5)	1 (25)	9 (33.3)	12 (19.4)
Macules	**–**	–	1 (3.7)	1 (1.6)
Papules	1 (3.2)	–	1 (3.7)	2 (3.2)
Maculopapular rash	**–**	**–**	3 (11.1)	3 (4.8)
Hyperkeratosis	**–**	–	2 (7.5)	2 (3.2)

– Not applicable.

**Fig. 2:**
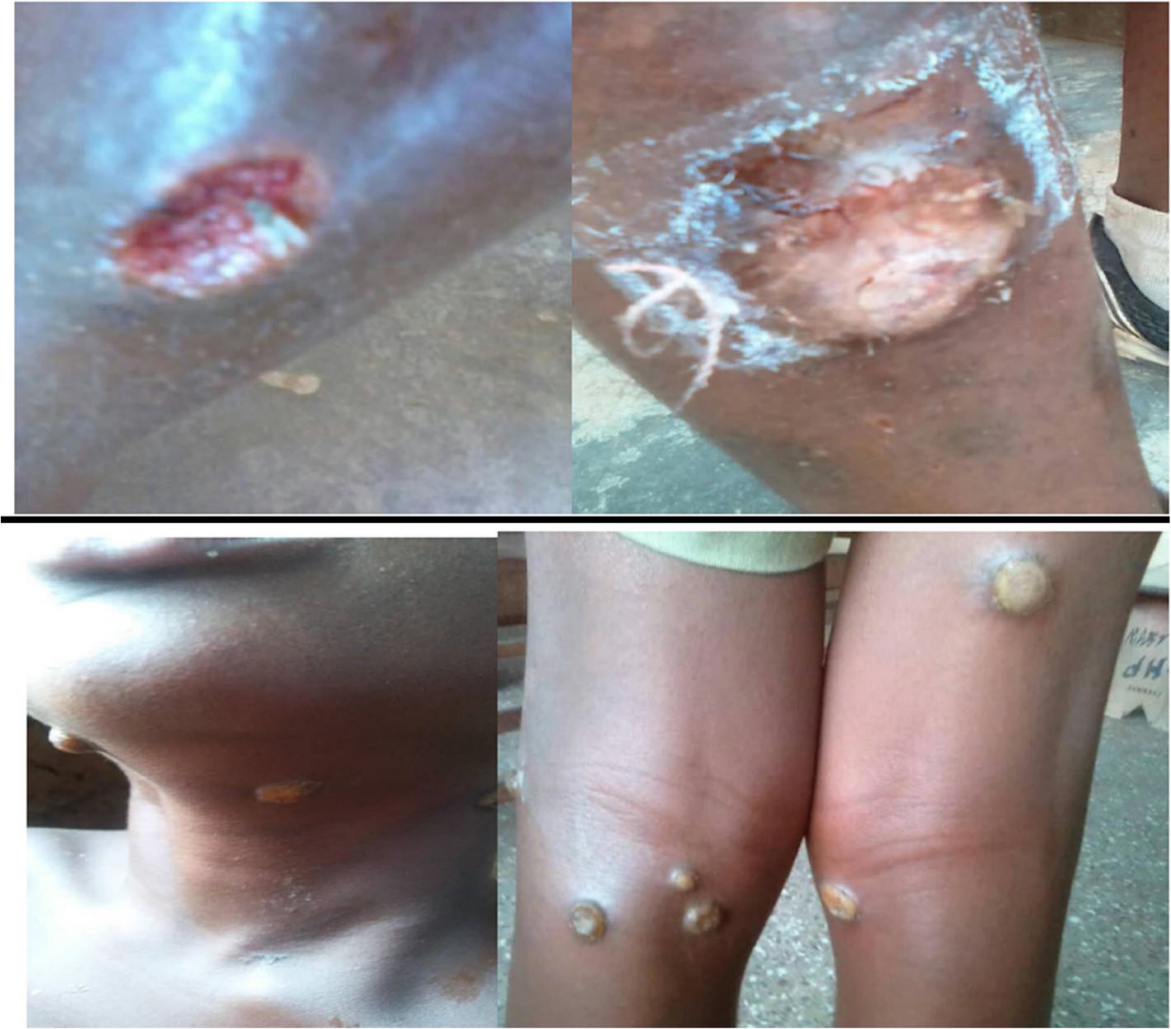
Upper: Yaws ulcers showing the pathognomonic “punched out” edges; Lower: Multiple papilloma on the neck and lower limbs of two study participants

Majority of the lesions were ulcers (67%, 42/62). The second most frequent lesion was papilloma (19.4%, 12/62). The other types of lesions; papules, macules, hyperkeratosis and maculopapular rash were less frequent contributing to less than 5% of the total number of cases seen (Table 2 and Fig. 2).

### Socio-demographic factors associated with yaws transmission

There was a significant association between sex and yaws infection. Yaws cases were two times more likely to be males (c*OR* = 2.70, 95% *CI:* 1.38–5.26). The Ga/Adangme (c*OR* = 4.80, 95% *CI:* 1.23–18.63) and Ewe (c*OR* = 4.95, 95% *CI:* 1.31–18.66) ethnic groups had increased odds of yaws infection compared to the other ethnic groups (Table 3).

**Table 3:** Summary of univariate analysis - Factors significantly associated with yaws (All cases and confirmed cases)

Variables	All cases		Confirmed cases	
	c*OR* (95% *CI*)	*P*-value	c*OR* (95% *CI*)	*P*-value
Socio-demographic factors				
Sex (Male)	2.70 (1.38–5.26)	0.004	2.70 (1.12–6.49)	0.027
Ethnicity (Ga/Adangme)	4.80 (1.23–18.63)	0.023	NS	
Ethnicity (Ewes)	4.95 (1.31–18.66)	0.018	9.68 (1.18–79.09)	0.034
Past treatment for yaws (Yes)	2.7 (1.13–6.47)	0.025	3.48 (1.26–9.60)	0.016
Behavioral factors				
Bath every day (No)	2.76 (1.31–5.80)	0.008	4.50 (1.87–10.84)	0.001
Frequency of bathing/day (Once)	1.98 (1.07–3.68)	0.03	3.50 (1.49–8.21)	0.004
Sharing of towels/sponge (Yes)	3.10 (1.40–6.97)	0.005	3.50 (1.17–10.94)	0.025
Bathroom (used by > 5 persons)	2.57 (1.36–4.80)	0.003	NS	
Contact with yaws (Yes)	2.70 (1.42–5.11)	0.002	5.23 (2.26–12.09)	< 0.0001
Use of clothing (Not regularly)	2.76 (1.15–6.59)	0.022	NS	
Handwashing (Not regularly)	4.98 (2.42–10.27)	< 0.0001	4.10 (1.65–10.22)	0.002
Environmental factors				
Type of housing (Compound^a^)	2.91 (1.53–5.50)	0.001	4.74 (1.90–11.84)	0.001
Sleeping room occupancy (> 4 persons)	3.31 (1.71–6.41)	< 0.001	4.5 (1.20–10.49)	< 0.0001
House occupancy (> 8 persons)	6.78 (2.24–20.50)	0.001	6.22 (1.57–24.63)	0.009
Toilet facility (open defecation)	2.70 (1.25–6.16)	0.012	3.70 (1.37–10.04)	0.01

c*OR* Crude odds ratio, *CI* Confidence interval, *NS* No statistical significance; ^a^ compound house – housing structure with multiple households (> 2) with shared facilities (cooking, bathing, toilets)

### Behavioral factors associated with yaws transmission

Sharing of personal items such as towels and sponge (c*OR* = 3.10, 95% *CI:* 1.40–6.97) and sharing a bathroom with more than five persons (c*OR* = 2.57, 95% *CI:* 1.36–4.80) were associated with active yaws infection. A previous contact with someone who had yaws was associated with yaws infection. Compared to community controls, yaws cases had increased odds of previous exposure to the disease (c*OR* = 2.70, 95% *CI:* 1.42–5.11). Comparatively, cases were less likely to be fully clothed or dressed regularly (c*OR* = 2.76, 95% *CI:* 1.15–6.59). Good hand washing practices was less frequent among cases compared to community controls. A greater proportion of cases compared to controls (c*OR* = 4.98, 95% *CI:* 2.42–10.27) reported that they did not wash their hands regularly when required (Table 3).

### Environmental factors associated with yaws transmission

Yaws cases had increased odds of staying in a compound house compared to controls. Compared to community controls, cases had a three-fold increased odds of sharing a sleeping room with more than four persons (c*OR* = 3.31, 95% *CI:* 1.71–6.41) or sharing a house with 5–8 persons (c*OR* = 4.65, 95% *CI:* 1.68–12.86) or more than eight persons (c*OR* = 6.78, 95% *CI:* 2.24–20.50) (Table 3).

The factors associated with yaws from the adjusted analysis were residence in a compound house (a*OR* = 25.42, 95% *CI:* 6.15–105.09), age (a*OR* = 5.90, 95% *CI:* 1.97–17.67), male sex (a*OR* = 4.15, 95% *CI:* 1.29–13.36), sharing a bathroom with more than five persons (a*OR* = 3.25, 95% *CI:* 1.09–9.71) and poor hand washing habits (a*OR* = 6.46, 95% *CI:* 1.89–22.04) (Table 4). Table 5 summarizes factors that were not significantly associated with yaws transmission.

**Table 4:** Multivariate analysis of factors associated with yaws (all cases) in the Upper West Akyem and Awutu Senya West districts

Variables	a*OR*	95% *CI*	*P*-value
Age (years)	5.90	1.97–17.67	0.002^ǂ^
Sex (Male)	4.15	1.29–13.36	0.039^ǂ^
Handwashing (Not regularly)	6.46	1.89–22.04	0.003^ǂ^
Number of persons sharing a bathroom (> 5 persons)	3.25	1.09–9.71	0.035^ǂ^
Type of toilet facility (open defecation)	3.59	0.99–12.99	0.052
Type of housing (Compound house) ^a^	25.42	6.15–105.09	< 0.0001^ǂ^
Sleeping room occupancy (> 4 persons)	2.60	0.79–8.58	0.117

^ǂ^*P* < 0.05 means statistically significant; a*OR* Adjusted odds ratio; ^a^compound house – housing structure with multiple households (> 2) with shared facilities (cooking, bathing, toilets)

**Table 5:** Summary of univariate analysis- Factors not significantly associated with yaws (all cases)

Variables	c*OR* (95% *CI*)	*P*-value
Level of education of caregiver		
None	ref	–
Primary	1.1 (0.48–2.50)	0.83
Junior High School	0.92 (0.36–2.36)	0.866
Senior High School	1.05 (0.17–6.60)	0.962
Bathing with soap		
Yes	ref	–
No	0.99 (0.23–4.11)	0.99
Building material		
Cement block	ref	
Mud	1.06 (0.51–2.0)	0.832
Source of water for household activities		
Pipe borne water	–	–
Bore hole	0.58 (0.12–2.66)	0.484
Well	1.63 (0.55–4.78)	0.375
Streams/rivers/ponds	1.52 (0.59–3.96)	0.389
Distance from water source		
< 5 min	ref	
5–10 min	1.69 (0.60–4.27)	0.352
> 10 min	1.81 (0.68–4.85)	0.236

c*OR* Crude odds ratio, *CI* Confidence interval

## Discussion

Poor personal hygiene, overcrowding and lack of access to improved sanitary facilities are the major factors that facilitate the transmission of yaws in the Awutu Senya West and Upper West Akyem districts. This is partly enhanced by the poor living standards in endemic communities in both districts.

Previous case-control studies conducted in Ghana by Dzotsi and colleagues showed no significant association between age and yaws [20]. In this study however, the adjusted analysis showed that increasing age was associated with increased odds of infection. This is similar to findings by Marks et al. [13]. Their sero-prevalence study demonstrated that increasing age was significantly associated with active disease [13]. Exposure to activities that can lead to trauma and abrasions tend to increase with age more especially among males. This increases the risk of infection with the bacterium. The injury sites serve as conducive environments and portals that facilitate the transmission of yaws.

Previous infection with yaws does not result in long lasting immunity and individuals previously infected carry a significant risk of reinfection. This could possibly be as a result of reactivation of latent yaws in a previously untreated patient [6]. This study showed that yaws cases had a threefold increased odd of being previously infected. This confirms the fact that relapses of asymptomatic, infected individuals is a major factor that drives the reemergence of yaws particularly in communities that have benefited from control efforts [23]. The risk of reinfection is also enhanced by the fact that previously infected individuals continue to reside in highly endemic communities which puts them at an increased risk. Marks and colleagues demonstrated a strong association between the degree of village endemicity and active yaws infection [10].

Infected persons serve as important reservoirs for continuous transmission of yaws. Thus, contact with an infected person significantly increases the risk of infection. Asymptomatic individuals with latent yaws infection also serve as potential sources of the reintroduction of infection into the community [23]. In this study, yaws patients had almost five fold-increased odds of previous contact or exposure to yaws. Transmission of yaws occurs through contact with infected fluid from lesions. The univariable analysis showed that the risk of infection was higher among study participants who share personal items like towels and sponge or use bathrooms shared by more than four persons.

Lack of access to proper sanitation such as potable drinking water, appropriate waste disposal facilities and toilet facilities facilitate the transmission of yaws [24]. We also found an increased risk of yaws transmission among persons who do not wash their hands regularly. From our study, more than half of the participants indicated a source of water within 5 minutes from their place of residence. This suggest that water is available to them. The worrying observation, therefore, is the fact that about eight in every ten cases admittedly do not wash their hands with soap. This brings to light the inadequacy of behavior change component in water sanitation and hygiene (WASH) programs being implemented. In recent times, efforts have been made to foster collaboration between WASH and NTD control programs for desired impacts. Key among them is the meeting involving WASH and NTD experts which was organized by Emory University in the USA and the Task Force for Global Health. The group agreed on a common vision, namely “Disease-free communities that have adequate and equitable access to water and sanitation, and that practice good hygiene” [25]. It is therefore important that this commitment is backed by implementation strategies with the well-crafted and well-tailored behavioral change effort.

The observation that persons living in compound houses are at increased risk of yaws is consistent with other studies. It is well established that persons living in compound houses belong largely to the low-socioeconomic groups and in most instances, are overcrowded. It is also evident from the literature that yaws thrive in socio-economically disadvantaged communities where overcrowding is common. According to Mackey and colleagues, staying in a compound house, sharing a sleeping room with more than four persons or staying in an overcrowded house significantly increases the risk of yaws transmission [24]. This is attributed to the fact that the mode of yaws transmission is through skin contact with an infected person. In compound houses, there is high interaction amongst the people and therefore, the risk of transmission of yaws can occur when individuals with active yaws lesions come into close contact with other people by sharing bathrooms and or sleeping together as was observed in our study. In a cluster randomized study by Marks et al., in the Solomon Islands, *Treponema pallidum* particle agglutination assay (TPPA) positivity and dual sero-positivity were associated with increased number of dual positive household contacts [13]. Similar studies have also found this significant association of yaws and environmental factors such as overcrowding [20, 26–28].

The choice of case-control study design could amount to recall bias particularly among the controls. Also, swabs from yaws-like ulcers in some endemic areas have shown infections with *Haemophilus ducreyi* alone or co-infections with *T. pallidum* subspecies *pertenue* and *H. ducreyi* [9, 11, 16, 17, 29]. The prevalence of sub-standard sanitary and personal hygienic conditions in yaws-endemic communities also facilitates the persistence of *H. ducreyi* infections in these communities [29]. The presence of *H. ducreyi* in lesions was however not determined in this study. Finally, prevalence-incidence bias is encountered in case control studies where exposures may rather tend to be protective typically when the disease involved is chronic. To reduce this, incident cases (active disease) were included in the study.

## Conclusions

Poor personal hygiene, overcrowding and lack of access to improved sanitary facilities are the factors associated with the transmission of yaws in the Awutu Senya West and Upper West Akyem districts. The skin lesions were also more common among males and school-aged children. Improving living conditions, access to good sanitary facilities and encouraging good personal hygiene practices should be core features of eradication programs in endemic communities.

## Data Availability

The data collected for the study which has been analyzed and presented are available at the first authors’ institution and is available upon formal request.

## Abbreviations

DPP: Dual Path Platform
GHS-ERC: Ghana Health Service Ethics Review Committee
NTD: Neglected tropical disease
NYEP: National Yaws Eradication Program
POC: Point of care
RDT: Rapid diagnostic test
WASH: Water sanitation and hygiene
WHO: World Health Organisation
